# Characteristic Mean Kurtosis Values in Simple Diffusion Kurtosis Imaging of Dentigerous Cysts

**DOI:** 10.3390/diagnostics13243619

**Published:** 2023-12-07

**Authors:** Yuka Fukumura, Masahiro Kuroda, Suzuka Yoshida, Yoshihide Nakamura, Yuki Nakamitsu, Wlla E. Al-Hammad, Kazuhiro Kuroda, Ryo Kamizaki, Yudai Shimizu, Yoshinori Tanabe, Kohei Sugimoto, Masataka Oita, Irfan Sugianto, Majd Barham, Nouha Tekiki, Nurul N. Kamaruddin, Yoshinobu Yanagi, Junichi Asaumi

**Affiliations:** 1 Department of Oral and Maxillofacial Radiology, Graduate School of Medicine, Dentistry and Pharmaceutical Sciences, Okayama University, Okayama 700-8558, Japan; pote5ytg@s.okayama-u.ac.jp (Y.F.);; 2 Radiological Technology, Graduate School of Health Sciences, Okayama University, Okayama 700-8558, Japan; 3 Department of Oral Medicine and Oral Surgery, Faculty of Dentistry, Jordan University of Science and Technology, Irbid 22110, Jordan; 4 Department of Health and Welfare Science, Graduate School of Health and Welfare Science, Okayama Prefectural University, Okayama 719-1197, Japan; 5 Graduate School of Interdisciplinary Sciences and Engineering in Health Systems, Okayama University, Okayama 770-8558, Japan; 6 Department of Oral Radiology, Faculty of Dentistry, Hasanuddin University, Sulawesi 90245, Indonesia; 7 Department of Dentistry and Dental Surgery, College of Medicine and Health Sciences, An-Najah National University, Nablus 44839, Palestine; 8 Department of Oral Rehabilitation and Regenerative Medicine, Graduate School of Medicine, Dentistry and Pharmaceutical Sciences, Okayama University, Okayama 700-8558, Japan; 9 Department of Dental Materials, Faculty of dentistry, Hasanuddin University, Sulawesi 90245, Indonesia

**Keywords:** dentigerous cyst, mean kurtosis, simple diffusion kurtosis imaging, head and neck, magnetic resonance imaging, apparent diffusion coefficient value, diffusion kurtosis imaging

## Abstract

We evaluated the usefulness of simple diffusion kurtosis (SD) imaging, which was developed to generate diffusion kurtosis images simultaneously with an apparent diffusion coefficient (ADC) map for 27 cystic disease lesions in the head and neck region. The mean kurtosis (MK) and ADC values were calculated for the cystic space. The MK values were dentigerous cyst (DC): 0.74, odontogenic keratocyst (OKC): 0.86, ranula (R): 0.13, and mucous cyst (M): 0, and the ADC values were DC: 1364 × 10^−6^ mm^2^/s, OKC: 925 × 10^−6^ mm^2^/s, R: 2718 × 10^−6^ mm^2^/s, and M: 2686 × 10^−6^ mm^2^/s. The MK values of DC and OKC were significantly higher than those of R and M, whereas their ADC values were significantly lower. One reason for the characteristic signal values in diffusion-weighted images of DC may be related to content components such as fibrous tissue and exudate cells. When imaging cystic disease in the head and neck region using SD imaging, the maximum b-value setting at the time of imaging should be limited to approximately 1200 s/mm^2^ for accurate MK value calculation. This study is the first to show that the MK values of DC are characteristically higher than those of other cysts.

## 1. Introduction

The application of magnetic resonance imaging (MRI) to head and neck diseases is increasing, and its effectiveness in diagnosing these diseases is garnering interest. Currently, apparent diffusion coefficient (ADC) values calculated using diffusion-weighted (DW) images and their ADC maps are widely used in routine clinical practice; however, restricted diffusion-weighted (RD) images are expected to be more useful, prompting many clinical studies [1,2,3,4,5].

Diffusion kurtosis (DK) imaging, a type of RD imaging, provides the microscopic structure of biologic tissues. Clinical studies using DK imaging has been conducted and its effectiveness has been reported [6,7]. However, DK imaging requires a long imaging time because the method initially presented involves imaging with many b-values in 30 axes [8,9,10]. Furthermore, software for creating DK images is not widely available in routine clinical practice. 

Hamada et al. first reported the development of a new imaging software for clinical applications and a simple method to create RD images [11]. In addition, Kuroda et al. [12] reduced the maximum b-value and shortened the imaging time, thereby reducing image distortion caused by body movement in the head and neck region. Because the tissues of the head and neck are nearly isotropic and have few diffusion limitations, they attempted to create DK images using 3-axis DW images, which are commonly used in routine clinical practice [12]. Using the simple diffusion kurtosis (SD) imaging [12] developed in this process, we were able to compare the mean kurtosis (MK) and ADC values at the pixel level by simultaneously acquiring the DK image and creating the ADC map. Recently, we reported the usefulness of SD imaging for diagnosing head and neck malignancies [13]; however, there are no reports on the use of SD imaging in other diseases.

In DW images of cystic diseases, odontogenic keratocysts (OKC) showed characteristic MK and ADC values [5]. Dentigerous cysts (DC) have also been reported to have characteristic ADC values [1,2,4]; however, there are no reports on their MK values.

In this study, we report the MK values of DC, which were found to indicate characteristic signal values using SD imaging, and discuss the literature, focusing on the fact that the signal values in cysts are derived from their content components. In addition, the optimal b-value setting for SD imaging in diagnosing cysts is discussed.

This is the first clinical report to demonstrate the diagnostic utility of DK and SD imaging of DC.

## 2. Materials and Methods

### 2.1. Patients

This study included 55 patients with cystic disease lesions who underwent head and neck MRI examinations as part of routine medical care between 25 March 2020 and 1 November 2022. The twenty-nine patients with hard tissue disease, a short diameter of the mass (<10 mm), and artifacts in the lesion images were excluded in the selection of select head and neck cysts. The cases were diagnosed pathologically by surgery or biopsy, or by characteristic clinical and imaging findings. Twenty-seven head and neck cysts in 26 patients were evaluated in this retrospective study. All patients provided written informed consent for undergoing MRI. The study was conducted in accordance with the guidelines of the Declaration of Helsinki and was approved by the Ethics Committee of the Okayama University Graduate School of Medicine, Dentistry and Pharmaceutical Sciences and the Okayama University Hospital, 2209-014.

### 2.2. MRI and Sequence

The MRI machines used were 3T MAGNETOM Prisma, 3T MAGNETOM Verio, 3T MAGNETOM Skyra, and 1.5T MAGNETOM Aera (Siemens Healthcare, Erlangen, Germany) with head and neck coils. DW images with 3 axes and three b-values were obtained using imaging conditions that are used for imaging ADC maps in routine clinical practice. The representative parameters were as follows: three b-values (0, 400, and 800 s/mm^2^); slice thickness = 3 mm; repetition time (TR)/time to echo (TE) = 6990–12300/55–84 msec; field of view (FOV) = 200 mm × 200 mm; gap = 4 mm; matrix = 140 × 140, 128 × 128, 126 × 126; and bandwidth = 990 Hz/pixel. The average imaging time for DW images was 390 s. In addition to the DW images, contrast-enhanced T1-weighted, T1-weighted, T2-weighted, and T2-STIR-weighted images were obtained as part of routine clinical practice.

### 2.3. Creation of the SD Image and ADC Map

We used a previously reported SD imaging method [11,12,13]. Specifically, using the three b-value DW images used to create the ADC map in routine clinical practice, we employed the developed software [11,12] to create a calculated image of the MK values, which we will subsequently refer to as the SD image herein. In this method, the image analysis software ImageJ 1.51h (U. S. National Institutes of Health, Bethesda, MD, USA) and Microsoft Excel 2019 (Microsoft, Redmond, WA, USA) macro programs were used to create an SD image simultaneously with an ADC map, using multiple b-value DW images.

Specifically, in each pixel of the three DW images with b-values of 0, 400, and 800 s/mm^2^, each signal value was logarithmically converted on the vertical Y-axis and the b-value on the horizontal X-axis. The quadratic function y = Ax^2^ + Bx + C is approximated to obtain the quadratic coefficient A and linear coefficient B, and Equation (1) is used to compute the MK value for each pixel. ImageJ converts MK values into images and creates SD images [11,12].

MK = 6A/(−B)^2^ (1)

In creating the ADC map, in each pixel of the three DW images with b-values of 0, 400, and 800 s/mm^2^, each signal value was logarithmically converted to a linear function y = Ax + B on the vertical Y-axis and the b-value on the horizontal X-axis. The linear coefficient A is obtained by approximating the linear function y = Ax + B. The ADC value of each pixel was calculated as -A and imaged using ImageJ to create an ADC map.

### 2.4. Region of Interest (ROI) Settings

The ROI was set by the consensus of five radiologists (M.K., J.A., Y.S., Y.F., and S.Y. with 39, 26, 6, 4, and 2 years of experience, respectively, in diagnostic imaging). DW images with b-values of 0 s/mm^2^ were used to determine the location and shape of the cysts; however, T2-STIR was used as a reference if necessary. In the present study, ROIs were set up along the inside of the cyst wall to evaluate the signal values in the cystic space.

### 2.5. Statistical Analysis

The MK and ADC values of all pixels in the ROI of the cystic area were extracted and analyzed for each case. A comparison of each histological type was performed for DC, OKC, ranulas (R), and mucous cysts (M), consisting of multiple cases. For comparison between the four tissues (DC, OKC, R, and M), the signal values of all pixels in the ROI of the cyst area for each case were analyzed together in one analysis for each histological type; this is called pixel analysis. Kruskal–Wallis and Dunn tests were used to compare the signal values of each cyst tissue in all DK images and ADC maps. For comparison of the pixel number in the ROI of the cyst area for each case between the four tissues (DC, OKC, R, and M), the permutation test and Levene’s test were used to test for equal medians and variances in all DK images and ADC maps. To evaluate the discriminatory ability of cysts among different histological types, receiver-operating characteristic (ROC) analysis was performed for each MK and ADC value, and the cutoff values and area under the curve (AUC) were calculated. In ROC analysis, AUC values of 1.0–0.9, 0.9–0.8, 0.8–0.7, 0.7–0.6, and 0.6–0.5 were judged as “excellent”, “very good”, “good”, “satisfactory”, and “unsatisfactory”, respectively. The cutoff value was set as the point closest to the upper-left corner. To examine the signal values of cysts in DW images for each histological type, the mean logarithm of the signal values of the ROI in the cyst area on DW images for each b-value was calculated. The b-value of the DW image on the horizontal axis and the mean value on the vertical axis were plotted for each histological type, and the quadratic and linear coefficients of the quadratic approximation curves were evaluated. Statistical analyses were performed using R (v4.2.2; URL: https://www.r-project.org/, accessed on 16 September 2022), EZR (v1.61; URL: https://www.jichi.ac.jp/saitama-sct/SaitamaHP.files/statmedEN.html, accessed on 16 September 2022), and SPSS (v27.0, IBM Corp., Armonk, NY, USA). A *p*-value < 0.05 was considered a statistically significant difference.

## 3. Results

After applying the exclusion criteria to the indications for case selection, 27 cases of head and neck cysts (four DC, six OKC, six R, eight M, one radicular cyst, one nasoalveolar cyst, and one postoperative maxillary cyst) in 26 patients (mean age: 48, range: 9–92 years, 17 males and 9 females) were finally included in the study (Table 1). As shown in Table 1, pathologically confirmed diagnoses were obtained by surgery or biopsy in 13 of the 27 cases: four DC, six OKC, one R, one radicular cyst, and one nasoalveolar cyst. The remaining 14 cases were confirmed based on characteristic clinical and imaging findings. In the pathological diagnosis, four cases of DC showed fibrous connective tissue and inflammatory cell infiltration in all cysts, whereas, in six cases of OKC, the cyst wall was composed of fibrous connective tissue, and keratinizing material was observed in the cystic space.

Table 1 summarizes the MK and ADC values in the DK image and ADC map for each case; the MK value for DC was higher than that for R and M in most cases, while the ADC value was lower. The same trend was observed for OKC. When comparing the MK and ADC values for the same tissue type, the MK values tended to show greater variability.

**Figure 1:**
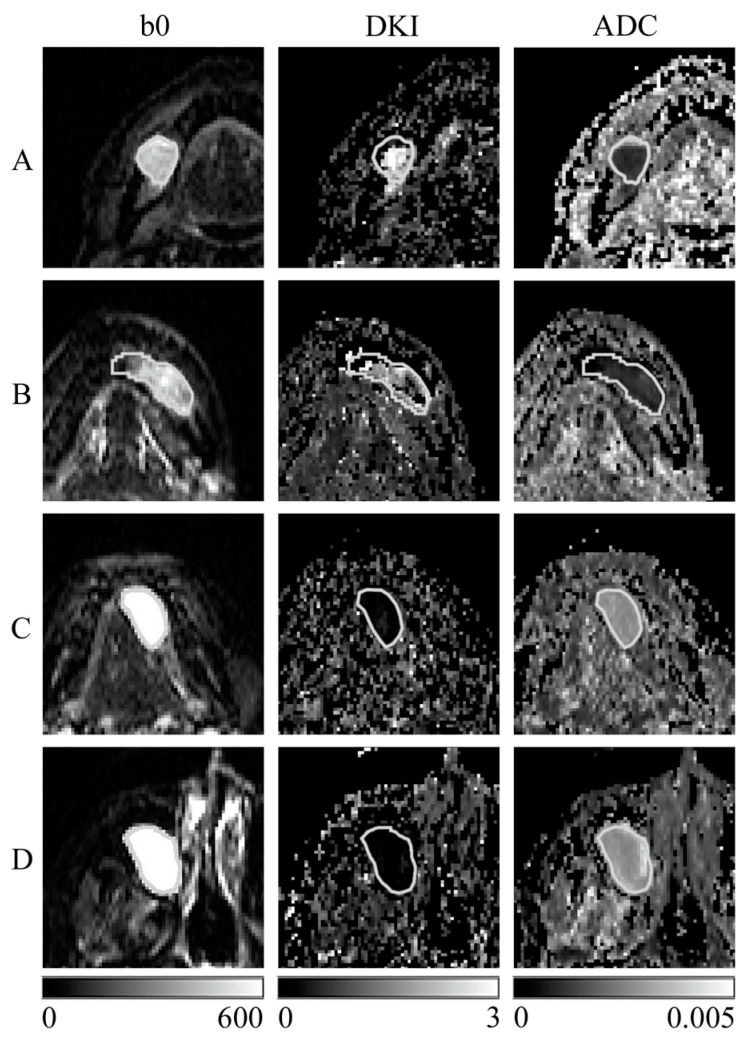
Representative images of each tissue sample are shown. (**A**) Dentigerous cyst; (**B**) odontogenic keratocyst; (**C**) ranula; (**D**) mucous cyst; (b0) diffusion-weighted (DW) images with a b-value of 0 s/mm^2^; (DKI) diffusion kurtosis (DK) imaging; and (ADC) apparent diffusion coefficient (ADC) map created with DK imaging using the same DW images with b-value 0, 400, and 800 s/mm^2^. The white line indicates cystic ROI. Scale bars indicate the displayed range of signal values for each image: DW image (0–600), DK imaging (0–3), and ADC map (0–0.005 mm^2^/s).

Figure 1 shows images of representative cases with an ROI. DC and OKC had lower signal values in DW images with a b-value of 0 s/mm^2^, higher MK values in DK images, and lower ADC values in ADC maps than R and M. The DK images of DC and OKC were more heterogeneous than the ADC maps.

**Figure 2:**
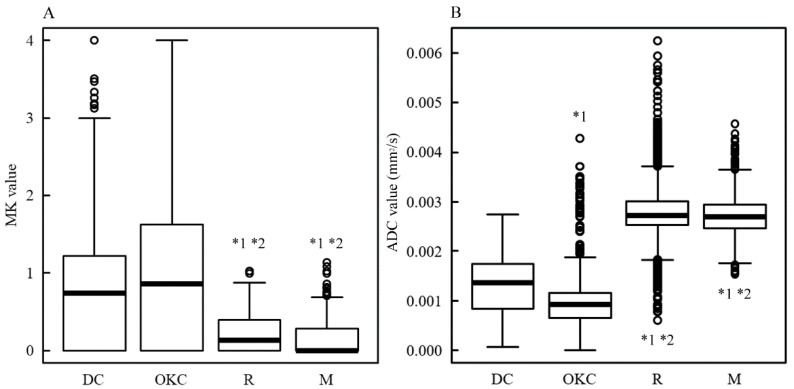
The box and whisker plot of the MK and ADC values. The vertical axis indicates the MK value (**A**) and ADC value (**B**) of the dentigerous cyst (DC), odontogenic keratocyst (OKC), radicular cyst (R), and mucous cyst (M). The horizontal bold line in each box is a median (50th percentile) of the measured values, the top and bottom of the boxes represent 25th and 75th percentiles, respectively, and whiskers indicate the range from the largest to smallest observed data points within the 1.5 interquartile range presented by the box. Circles indicate outliers. *p*-values were calculated via the Kruskal–Wallis test. *1 and *2 indicate a significant difference of *p* < 0.001 against DC and OKC, respectively.

In Table 1, the mean and standard deviation of the number of pixels in the ROI for each case was 101 ± 65 for histological types DC, OKC, R, and M, which consisted of multiple cases. In the permutation and Levene tests, DC and OKC did not differ significantly from R and M in terms of the number of pixels or their variance. The difference between DC and OKC was not significant. Therefore, pixel analysis was performed for DC, OKC, R, and M, as shown in Figure 2.

Figure 2 shows box and whisker plots of the MK (Figure 2A) and ADC (Figure 2B) values from the pixel analysis for DC, OKC, R, and M. The median MK values (lower quartile, upper quartile) were DC: 0.74 (0, 1.22), OKC: 0.86 (0, 1.62), R: 0.13 (0, 0.40), and M: 0 (0, 0.28); the median ADC values were DC: 1364 (847, 1746) × 10^−6^ mm^2^/s, OKC: 925 (663, 1166) × 10^−6^ mm^2^/s, R: 2718 (2537, 3010) × 10^−6^ mm^2^/s, and M: 2686 (2466, 2941) × 10^−6^ mm^2^/s. The results of the Kruskal–Wallis test showed that both DC and OKC had significantly higher MK values and significantly lower ADC values than R and M (*p* < 0.001). The ADC value for OKC was significantly lower than that for DC. The MK value for OKC was higher than that for DC; however, the difference was insignificant. 

**Figure 3:**
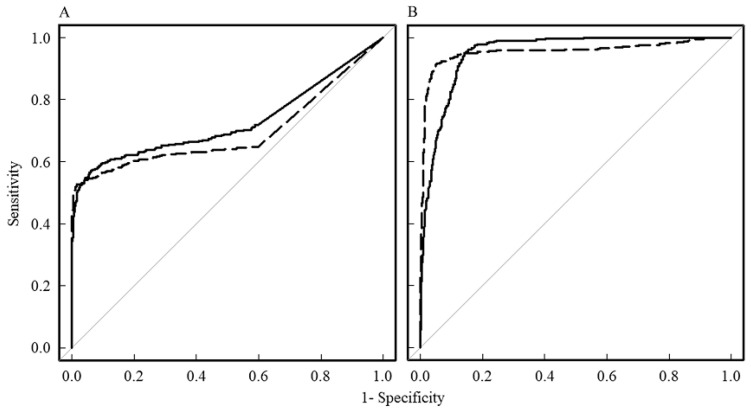
ROC curves against ranula based on pixel analysis. The ROC curves for mean kurtosis (MK) and apparent diffusion coefficient (ADC) values are shown in (**A**,**B**), respectively. The solid lines and dotted lines indicate the ROC curves for dentigerous cyst (DC) and odontogenic keratocyst (OKC), respectively.

Figure 3 shows the results of the ROC analysis against R by pixel analysis for the MK (Figure 3A) and ADC (Figure 3B) values. The ROC curves for DC and OKC are shown against R. For discrimination between DC and R, the AUC for MK was 0.725, the test quality was “good”, and the cutoff value was 0.63. For discrimination between OKC and R, the AUC for MK was 0.693, the test quality was “satisfactory”, and the cutoff value was 0.74. The AUC for the ADC of DC was 0.952, the test quality was “excellent”, and the cutoff value was 2204 × 10^−6^ mm^2^/s. The AUC for the ADC of OKC was 0.956, the test quality was “excellent”, and the cutoff value was 1664 × 10^−6^ mm^2^/s. The ROC curves for the MK and ADC values for both DC and OKC were significantly different (*p* < 0.001). There was no significant difference between the two ROC curves for DC and OKC for the MK and ADC values. 

**Figure 4:**
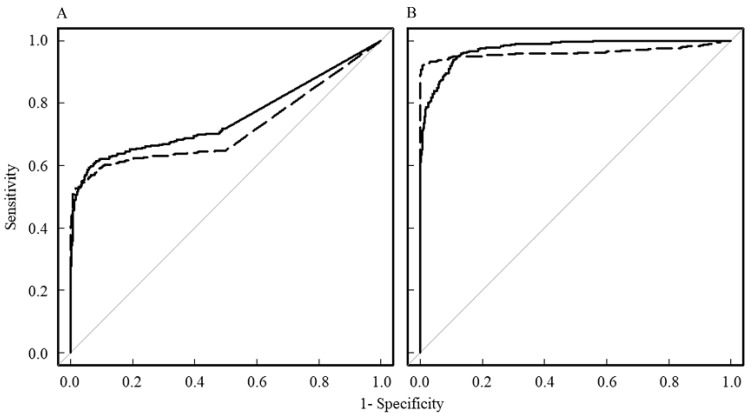
ROC curves against mucous cyst based on pixel analysis. The ROC curves for mean kurtosis (MK) and apparent diffusion coefficient (ADC) values are shown in (**A**,**B**), respectively. The solid lines and dotted lines indicate the ROC curves for dentigerous cyst (DC) and odontogenic keratocyst (OKC), respectively.

Figure 4 shows the results of the ROC analysis against M by pixel analysis for the MK (Figure 4A) and ADC (Figure 4B) values. The ROC curves for DC and OKC are shown against M. For discrimination between DC and M, the AUC for MK was 0.752, the test quality was “good”, and the cutoff value was 0.58. For discrimination between OKC and R, the AUC for MK was 0.718, the test quality was “good”, and the cutoff value was 0.76. The AUC for the ADC of DC was 0.975, the test quality was “excellent”, and the cutoff value was 2143 × 10^−6^ mm^2^/s. The AUC for the ADC of OKC was 0.963, the test quality was “excellent”, and the cutoff value was 1724 × 10^−6^ mm^2^/s. The ROC curves for the MK and ADC values for both DC and OKC were significantly different (*p* < 0.001). There was no significant difference between the two ROC curves for DC and OKC for the MK and ADC values.

**Figure 5:**
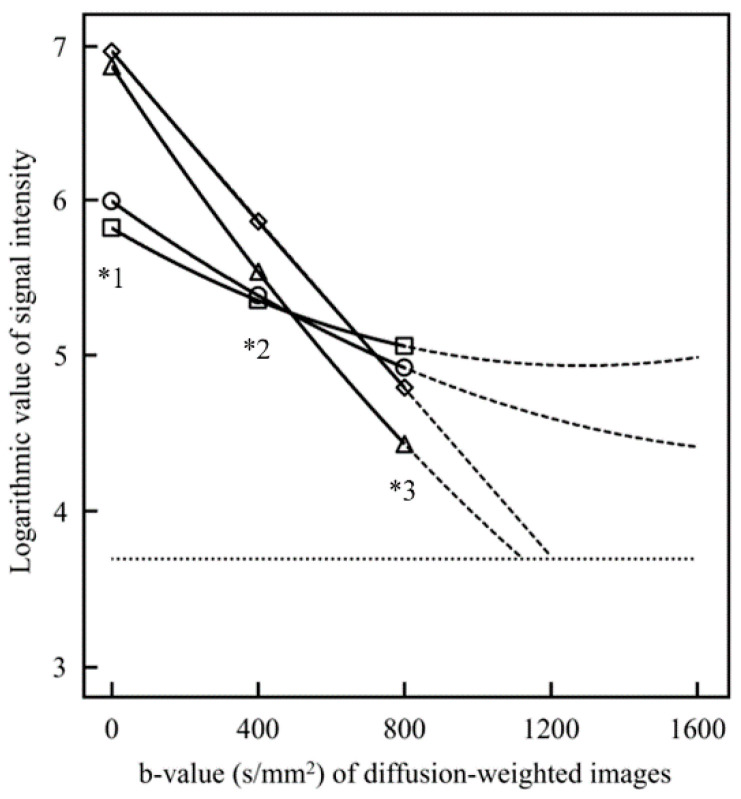
Signal intensity of the diffusion-weighted (DW) image of each cyst. The vertical axis of the graph shows the logarithmic value of the signal intensity of the DW image. The horizontal axis shows the b-values (s/mm^2^) of each DW image. Dentigerous cyst: open circle, odontogenic keratocyst: open square, ranula: open triangle, and mucous cyst: open diamond. The dotted curve is the backward prediction by backward extension of the quadratic approximation curve above the b-value of 800 s/mm^2^. The dotted horizontal line indicates that the DW image is the logarithm of the signal value of 40 for the noise level. *1 and *3 indicate a significant difference of *p* < 0.01 among DC, OKC, R, and M of b-values 0 and 800 s/mm^2^. *2 indicates a significant difference of *p* < 0.01 between DC-R, DC-M, OKC-R, OKC-M, and R-M of b-value 400 s/mm^2^.

Figure 5 shows the signal values for each cyst on DW images. The relationship between the b-value of the DW image and the average logarithmic signal value in the ROI for each cyst type was plotted graphically. Significant differences (*p* < 0.01) in signal values were found in the DW images with b-values of 0 and 800 s/mm^2^ among the DC, OKC, R, and M tissues. The quadratic coefficients or magnitude of bending of the approximate curves of the graphs were DC: 4.41 × 10^−7^, OKC: 5.37 × 10^−7^, R: 6.72 × 10^−7^, and M: 8.34 × 10^−8^. The linear coefficients or decrease in signal values for increasing b-values were DC: −1.69 × 10^−3^, OKC: −1.38 × 10^−3^, R: −3.58 × 10^−3^, and M: −2.78 × 10^−3^. The slopes for DC and OKC were lower than those for R and M. The backward prediction of quadratic approximate curves with measured data at b-values of 0, 400, and 800 s/mm^2^ showed that the signal values in the DW images of R and M decreased to noise levels at approximately b-value 1200 s/mm^2^.

## 4. Discussion

Using our SD imaging technique, we clarified the characteristics of the MK and ADC values of cystic diseases in the head and neck regions. Our study is the first to derive the MK values of DC, indicating high MK values compared to those of general cysts. Previous studies have reported high MK values for OKC, with similar results using SD imaging. In this study, we examined the relationship between the MK values of DC and OKC and the signal values of DW images to determine the cause of high MK values and low ADC values, suggesting that differences in the components of cysts and tissues are involved.

A previous study reported the MK value of malignant sinonasal masses as 0.91 ± 0.23 [14], and those of the oropharynx and oral cavity were reported as 0.84 ± 0.19 [15]. In this study, the MK value (lower quartile, upper quartile) of DC, 0.74 (0, 1.22), was high for normal cysts such as R, 0.13 (0, 0.40) and M, 0 (0, 0.28) and showed MK values similar to those of tumors. A previous study reported the ADC values of DC as 978–1670 × 10^−6^ mm^2^/s [1,2,4], which were low for normal cysts and close to those of squamous cell carcinomas in the oral and maxillofacial region (1380 ± 220 × 10^−6^ mm^2^/s) [16]. We observed that the ADC values (lower quartile, upper quartile) of SD imaging, 1364 (847, 1746) × 10^−6^ mm^2^/s, were similar to those previously reported [1,2,4].

DC are benign odontogenic cysts that occur in a wide age range, with a peak frequency in the twenties and thirties; they are the second most common odontogenic cysts after radicular cysts, accounting for approximately 24% of all true cysts in the jaws [17,18,19]. DC develop from fluid accumulation between the reduced enamel epithelium of the follicle and the crown of an unerupted tooth, or between the layers of the reduced enamel epithelium. Some cases develop from inflammatory processes that occur at the root apex of the deciduous tooth and grow around the unerupted permanent tooth. If conventional radiography or CT shows a sclerotic bone structure around the cyst cavity, the cyst may contain inflammatory cells, necrotic cell debris, and fibrous or fibromyxoid tissue, thereby reducing the ADC value [19,20]. Furthermore, the contents of non-inflammatory DC and OKC include glycosaminoglycans, especially hyaluronic acid and proteins [19,21], which increase the viscosity of the contents and cause lower ADC values [2]. In the case of DC that develop from inflammatory processes, contamination with membrane components, such as cell debris, can limit the movement of water molecules and cause elevated MK values. The wide range of MK values for DC is thought to be due to differences in the developmental processes.

The mean MK value of OKC ± standard deviation (the 95% confidence interval) was reported to be 1.16 ± 0.33 (0.82–1.51) [5] when imaged at a maximum b-value of 1500 s/mm^2^, which was high compared to other benign cysts such as R (median MK value 0.13) and M (median MK value 0). The MK value with our SD imaging method using a maximum b-value of 800 s/mm^2^ was similar, regardless of the difference in the maximum b-value. The ADC value of OKC was reported to be 850–1030 × 10^−6^ mm^2^/s in previous studies [1,2,3,4,5], similar to the 925 (663, 1166) × 10^−6^ mm^2^/s derived in this study; in both studies, the ADC values of OKC were lower than those of other odontogenic cysts [1,2,3,4,5].

Histologically, OKC arises from the dental lamina and is composed of a cystic space containing desquamated keratin lined with a parakeratinized uniform squamous epithelium of 5–10 cell layers containing exfoliated keratin [22,23]. In OKC, increased viscosity due to hyaluronic acid [21] and contamination with desquamated keratin [20,24,25] cause lower ADC and higher MK values.

Most cystic lesions, except OKC and epidermoid cysts, which contain large amounts of internal keratinization, typically exhibit more hypointense T1-weighted images and hyperintense T2-weighted images than the superficial adipose tissue [26]. However, some foci in each image did not exhibit the aforementioned trends in signal intensity. Currently, MRI can help distinguish such foci from other cystic regions. In T2-weighted images of DC, the internal periapical area typically exhibits a homogeneous high signal but may also exhibit a heterogeneous signal [26]. OKC may show an increased T1 signal in the keratinaceous material regions and an increased T2/STIR signal in the fluid regions. Additionally, OKC may internally exhibit intermediate homogeneous T1 and T2 signals [26].

Because the DW image with a b-value of 0 s/mm^2^ is a T2-weighted image, the signal values of DC and OKC in the DW image with a b-value of 0 s/mm^2^ (Figure 1) were lower than those of other cysts, which, together with the reasons for the higher MK values in the DK image and lower ADC values in the ADC map, may be related to the characteristic components of the cyst.

Signal values in the DW images (Figure 4) decreased exponentially rather than linearly with increasing b-values; signal values for R and M with low MK values decreased with increasing b-value more than those for DC and OKC with high MK values. The stronger the slope of the line, the greater the degree of diffusion. The higher the b-value, the lower the signal value of water; thus, the signal is lost to the noise level. It is difficult to accurately measure the ADC and MK values in the noise level region at this high b-value. From Figure 4 in this study, we inferred that in R and M, the signal value becomes the noise level when the maximum b-value is approximately 1200 s/mm^2^. In DC and OKC, the signal value may not become a noise level until the maximum b-value is approximately 1600 s/mm^2^; however, the maximum b-value should be selected to be less susceptible to noise in actual imaging. Therefore, it is recommended that the maximum b-value be kept at approximately 1200 s/mm^2^ when differentiating cysts using MRI.

While ADC maps, which are image of free diffusion, are widely used in routine clinical practice, DK imaging, which images RD, requires a long imaging time and dedicated imaging software; therefore, it has not yet become widely used in routine clinical practice. Our SD imaging [12], using inexpensive software packages such as Excel and ImageJ, simultaneously created DK images and ADC maps. Since both the DK image and ADC map were created from the same source DW image, the MK value in the DK image and the ADC value in the ADC map could be compared pixel by pixel. Although the maximum b-value of 800 s/mm^2^ used in this study was too low for creating a DK image, Fusco et al. [27] reported that a maximum b-value of 800 s/mm^2^ is effective for the diagnosis of rectal cancer, and Quentin et al. [10] reported that a maximum b-value of 1000 s/mm^2^ is effective for the diagnosis of prostate cancer. Clinical studies using SD imaging have demonstrated its usefulness in diagnosing head and neck tumors [13]. Several previous reports [12,13] have reviewed the validity of the number of three axes used in SD imaging. Every measurement made in each direction of a voxel is unique and individual information, and increasing the number of directions would increase the accuracy of kurtosis. For each direction, a barrier such as a cell membrane determines its kurtosis value. The MK value is the average value of each of these kurtosis values. Compared to nerve tissues, which mainly have anisotropic diffusion, in tumor tissues, which mainly have isotropic diffusion, the kurtosis values from each direction are similar, and changes in the number of axes have little effect on MK values. As the number of axes increases, the number of kurtosis values from each direction used to calculate the MK value increases, and the information volume of signal increases, so the variance of the kurtosis values from each direction decreases, and the MK value improves accuracy. In addition, many clinical studies [12,28,29] have shown that a reduction in the number of axes has little effect on the MK values. There are many papers reporting the clinical usefulness of three-axis DK imaging, and their review by Rosenkrantz et al. [29] stated that three axis directions are sufficient as the number of imaging directions. As for the minimum b-value, its setting may affect the intravoxel incoherent motion (IVIM) effect. The IVIM effect may be added to the DW images of tissues with blood flow in the b-value range of 0–100 s/mm^2^. In general, as long as DW imaging of the b-value 0 s/mm^2^ is used, the IVIM effect will be included in MK values. To avoid the IVIM effect, the minimum b-value should be set to a low value, such as 100 s/mm^2^. However, since there is little blood flow, including perfusion, inside the cysts of interest in this study, and an IVIM effect inside the cyst might be unlikely, a minimum b-value of 0 s/mm^2^ was used.

One limitation of this study was the small number of cases and the limited number of lesion types. As a result, case selection bias may exist. Therefore, further studies with a larger number of cases and more lesion types are necessary to improve the reliability.

Another problem is the prominence of noise in the SD images. One reason for this is that the maximum b-value used for SD imaging was as low as 800 s/mm^2^ [13]. SD imaging uses 3-axis DW imaging, which may also be because the variation of the calculated signal values at each pixel in the acquired image is larger for 3-axis imaging than for 30-axis imaging [13]. Furthermore, the fact that SD imaging uses only three b-values may be one of the reasons for the large variations in MK values at each pixel [13]. To solve this problem, it is necessary to consider the development of filter processing for SD images to reduce the noise and variation inherent in SD imaging and optimize the imaging conditions.

## 5. Conclusions

In conclusion, this is the first study to characterize the MK values of DC using SD imaging, and the relationship between the causes of high MK and low ADC values and the signal values in DW images was investigated and considered to be related to the components of the cyst.

Although the maximum b-values in the DK imaging of cysts were set differently in previous studies, the MK and ADC values in this SD imaging with a low maximum b-value were almost the same as those reported in the past. SD imaging, which allows easy visualization of MK values, is expected to be useful in future clinical practice.

It is recommended to pursue future studies in the development of DK images of cysts.

## Figures and Tables

**Table 1:** Case information, MK value, and ADC value for each case. * Pixel number in the region of interest of the cyst set on the slice showing the maximum area of the cyst. Mean kurtosis value (MK). Apparent diffusion coefficient value (ADC). Lower quartile (Q1), median, and upper quartile (Q3) for MK and ADC.

Case	Histological Classification	Site	Pixel Number *	MK [Median (Q1, Q3)]	ADC [Median (Q1, Q3)] [×10^−6^ mm^2^/s]
1	Dentigerous cyst	Mandible	95	1.92 (0.40, 2.53)	781 (667, 965)
2			22	0.09 (0, 0.96)	953 (699, 1199)
3			81	0.49 (0, 0.83)	1633 (1373, 1886)
4			138	0.77 (0, 1.02)	1535 (1064, 1887)
5	Odontogenic keratocyst	Mandible	136	0.42 (0, 0.94)	891 (176, 1953)
6			58	1.20 (0, 1.64)	813 (724, 885)
7			69	0 (0, 0.79)	1049 (954, 1149)
8			200	1.35 (0, 1.89)	870 (656, 1051)
9			94	1.02 (0.61, 1.36)	1123 (843, 1356)
10			50	0.05 (0, 2.68)	619 (460, 1053)
11	Ranula	Oral floor	32	0 (0, 0.50)	1828 (1738, 2085)
12			146	0.40 (0.12, 0.54)	2009 (1611, 2412)
13			54	0.11 (0, 0.39)	4264 (3785, 4583)
14			144	0.04 (0, 0.20)	2717 (2594, 2833)
15			210	0.05 (0, 0.29)	2710 (2614, 2880)
16		Submandible	253	0.13 (0, 0.47)	2886 (2662, 3300)
17	Mucous cyst	Maxillary sinus	214	0 (0, 0.05)	2846 (2702, 3122)
18			45	0 (0, 0.28)	2830 (2621, 3052)
19			88	0 (0, 0.33)	2707 (2538, 2954)
20			113	0.05 (0, 0.29)	2713 (2555, 2986)
21			38	0.13 (0, 0.39)	2031 (1911, 2145)
22			20	0.45 (0, 0.64)	2046 (1914, 2258)
23			91	0.35 (0, 0.47)	2472 (2322, 2635)
24			28	0.05 (0, 0.35)	2225 (2029, 2333)
25	Radicular cyst	Maxilla	101	0 (0, 0.22)	2437 (2318, 2575)
26	Postoperative maxillary cyst	Maxillary sinus	40	0.87 (0.33, 1.23)	1247 (1095, 1365)
27	Nasoalveolar cyst	Nasolabial groove	53	0.50 (0, 0.77)	2280 (2077, 2603)

## Data Availability

The data presented in this study are available from the corresponding author upon reasonable request.
